# Topical KGF treatment as a therapeutic strategy for vaginal atrophy in a model of ovariectomized mice

**DOI:** 10.1111/jcmm.12334

**Published:** 2014-08-01

**Authors:** Simona Ceccarelli, Sirio D'Amici, Enrica Vescarelli, Paolo Coluccio, Pietro Matricardi, Cira di Gioia, Pierluigi Benedetti Panici, Ferdinando Romano, Luigi Frati, Antonio Angeloni, Cinzia Marchese

**Affiliations:** a Department of Experimental Medicine, Sapienza University of RomeRome, Italy; b Department of Public Health and Infectious Diseases, Sapienza University of RomeRome, Italy; c Servizio B.G.S.A–Settore Sperimentazione Animale, Istituto Superiore di SanitàRome, Italy; d Department of Drug Chemistry and Technologies, Sapienza University of RomeRome, Italy; e Department of Radiology, Oncology and Pathology, Sapienza University of RomeRome, Italy; f Department of Gynecologic-Obstetrical and Urologic Sciences, Sapienza University of RomeRome, Italy; g Department of Molecular Medicine, Sapienza University of RomeRome, Italy

**Keywords:** keratinocyte growth factor, vaginal atrophy, non-genomic ERα pathways

## Abstract

One of the most frequent complaints for post-menopausal women is vaginal atrophy, because of reduction in circulating oestrogens. Treatments based on local oestrogen administration have been questioned as topic oestrogens can reach the bloodstream, thus leading to consider their safety as controversial, especially for patients with a history of breast or endometrial cancers. Recently, growth factors have been shown to interact with the oestrogen pathway, but the mechanisms still need to be fully clarified. In this study, we investigated the effect of keratinocyte growth factor (KGF), a known mitogen for epithelial cells, on human vaginal mucosa cells, and its potential crosstalk with oestrogen pathways. We also tested the *in vivo* efficacy of KGF local administration on vaginal atrophy in a murine model. We demonstrated that KGF is able to induce proliferation of vaginal mucosa, and we gained insight on its mechanism of action by highlighting its contribution to switch ERα signalling towards non-genomic pathway. Moreover, we demonstrated that KGF restores vaginal trophism *in vivo* similarly to intravaginal oestrogenic preparations, without systemic effects. Therefore, we suggest a possible alternative therapy for vaginal atrophy devoid of the risks related to oestrogen-based treatments, and a patent (no. RM2012A000404) has been applied for this study.

## Introduction

Oestrogen deficiency at post-menopause causes thinning and shrinking of the vaginal epithelium, as well as decreased lubrication, with consequent inflammation of vagina and outer urinary tract in more than 50% of women [1]. The simultaneous reduction in glycogen in cells of the intermediate layer causes modifications of the vaginal ecosystem, with increased risk of recurrent vaginitis [2]. Vaginal atrophy can also occur in pre-menopausal women treated with adjuvant vaginal brachytherapy for endometrial cancer [3,4]. Therefore, these symptoms can be considered a significant health concern for a substantial portion of the female population, and are generally treated with systemic hormone replacement therapy (HRT). Nevertheless, the need for alternative methods to stimulate vaginal mucosa proliferation arises from the fact that systemic oestrogens can induce the development of urinary incontinence [5] and are involved in many pathological processes, such as breast and endometrial cancers [6,7]. Local oestrogen use is generally considered as safe, although some reports pointed out that oestrogen contained in intravaginal preparations is significantly absorbed and appears in the general circulation, thus indicating that its effects are not limited to the vagina, but can lead to systemic actions [8,9].

Oestrogen receptor α (ERα) exerts its function predominantly through binding to its ligand, 17β-Estradiol (E2), which induces receptor nuclear translocation and binding to oestrogen response elements (EREs) on DNA to regulate transcription of target genes (genomic or classical signalling) [10]. However, there is also a non-classical ERα signalling pathway that mediates some rapid effects of oestrogens that occur on a time scale of seconds to minutes [10,11]. This non-genomic signalling includes membrane-associated receptor activation and stimulation of cytoplasmic pathways, such as those of PI3K/Akt, ERK and p38 [12,13]. The factors that determine the choice between classical and non-classical pathway remain almost completely unknown.

In the last years, a number of studies demonstrated an ER-mediated activation of growth factors receptors, such as EGFR, HER2/Neu and IGFR [14,15], and it is known that the EGF/Ras/MAPK signalling pathway is able to activate ERα through phosphorylation at Ser118, as well as E2 [16].

Keratinocyte growth factor (KGF/FGF7), a member of the fibroblast growth factors (FGFs) family, acts by binding to the receptor tyrosine kinase FGFR2-IIIb/KGFR, generated through an alternative splicing of FGFR2 gene and predominantly expressed on epithelial cells of different organs [17]. KGF is essential for the maintenance of integrity and functionality of adult epithelial tissues, because of its cytoprotective and regenerative activities. In fact, its expression is strongly up-regulated upon injury in various epithelial tissues such as skin, kidney, bladder, pancreas, stomach, intestine and lung [18–20]. Treatment with recombinant KGF (Palifermin) is able to protect epithelial cells against a variety of injuries, including radiation-induced damage, and it has been approved by FDA for the treatment of severe oral mucositis that results from cancer radio and/or chemotherapy in patients with haematological or head and neck cancers [21,22]. On the other end, KGF administration in the developing vagina of mice during the neonatal period results in oestrogen-independent proliferation of the vaginal epithelium, thus suggesting a potential link between oestrogen treatment and activation of KGF/KGFR signalling [23,24].

In this study, we investigated the crosstalk between oestrogen and KGFR pathways, to clarify the mechanisms underlying the growth-promoting effect of oestrogens and KGF on vaginal mucosa cells. Moreover, we compared the *in vivo* efficacy of local oestrogen treatment with the topical administration of KGF, to propose this protein as a possible alternative therapy for post-menopausal vaginal atrophy or other dysfunctions, such those occurring in patients subjected to radiotherapy after endometrial cancer surgery. These results provide a novel insight for vaginal symptoms therapy, and a patent has been applied (no. RM2012A000404).

## Materials and methods

### Cell cultures and treatments

The ERα-positive MCF-7 human breast adenocarcinoma cell line, purchased from the American Type Culture Collection (No. HTB-22, ATCC-LGC Promochem, Teddington, UK), were cultured in DMEM (Invitrogen, Karlsruhe, Germany), supplemented with 10% foetal bovine serum (FBS; Invitrogen) and antibiotics. Primary cultures of human vaginal mucosa cells (HVMs) were established from 1-cm^2^ full-thickness biopsy of the vaginal mucosa, as previously reported [25], and maintained in Keratinocyte Basal Medium (Lonza Milano S.r.l., Milano, Italy) supplemented with KGM single quotes (Lonza Milano S.r.l., Milano, Italy), with medium change twice a week. Cells were treated with KGF (Upstate Biotechnology, Lake Placid, NY, USA), 17β-estradiol (E2; Sigma-Aldrich, Milan, Italy) or a combination of them for 24 hrs in proliferation experiments or for 5–30 min. in pathway analyses. Where indicated, cells were pre-treated with tamoxifen (Sigma-Aldrich, 100 nM) for 30 min. and then treated with KGF or E2 in the presence or absence of tamoxifen for 24 hrs. Prior to treatments, MCF-7 cells were grown in phenol red-free DMEM (Invitrogen) supplemented with 10% dextran charcoal-treated FBS (Invitrogen), and HVMs were switched to a steroid-reduced medium (KGM without EGF and BPE). Both MCF-7 and HVMs were also serum starved for 4 hrs.

### Quantitative real time PCR (qRT-PCR)

MCF-7 cells were harvested and total RNA was extracted with the use of TRIzol reagent (Invitrogen). cDNA was generated with oligo(dT) from 1 μg of RNA using the SuperScript III Reverse Transcriptase Kit (Invitrogen). After reverse transcription, the abundance of KGFR was quantified by qRT-PCR, as previously described [26], using 18S mRNA as endogenous control.

### Immunofluorescence microscopy

Cells, grown on coverslips, were fixed in 4% paraformaldehyde in PBS for 30 min. at 25°C, followed by treatment with 0.1 M glycine in PBS for 20 min. at 25°C and with 0.1% Triton X-100 in PBS for additional 5 min. at 25°C to allow permeabilization. Cells were incubated with anti-Ki67 rabbit polyclonal antibodies (1:50 in PBS; Zymed Laboratories, San Francisco, CA, USA), which identify cycling cells, followed by Texas Red conjugated goat anti-rabbit IgG (1:100 in PBS; Jackson ImmunoResearch Laboratories, West Grove, PA, USA). Nuclei were visualized using 4′,6-diamido-2-phenylindole dihydrochloride (1:10000 in PBS; Sigma-Aldrich). Fluorescence signals were analysed by recording stained images using a cooled CCD colour digital camera SPOT-2 (Diagnostic Instruments Incorporated, Sterling Heights, MI, USA) and Axiovision software (Carl Zeiss Inc., Oberkochen, Germany). The percentage of Ki67-positive cells was evaluated by counting, for each treatment, a total of 500 cells, randomly taken from 10 microscopic fields in three different experiments, expressed as mean ± SD and reported as graphs. For ERα localization experiments, actin cytoskeleton was visualized using TRITC–phalloidin (1:100 in PBS; Sigma-Aldrich) and ERα with an anti-ERα monoclonal antibody (1:100 in PBS; Santa Cruz Biotechnology, Santa Cruz, CA, USA). The single-stained and merged images were acquired with Zeiss Apotome® and Axiovision software (Carl Zeiss, Jena, Germany) using a 40× objective lens. Quantitative analysis was performed by counting cells exhibiting primarily nuclear localization of ERα (percentage of ERα-positive nuclei), or exhibiting membrane localization of ERα (percentage of ERα-positive membranes). More than 50 stained cells per microscopic field were counted. Results from three microscopic fields, expressed as mean ± SD, were reported in graphs.

### Cell survival assay

MCF-7 cells were fixed for 10 min. in a solution 10% acetic acid–10% methanol, stained with crystal violet (1% w/v) and photographed using a Power Shot G5 digital camera (Canon, Inc., Tokyo, Japan).

### Western blot analysis

Cells were lysed in RIPA buffer. Total proteins (50–150 μg) were resolved under reducing conditions by 7–10% SDS-PAGE and transferred to Immobilon-FL membranes (Merck Millipore, Billerica, MA, USA). Membranes were blocked in TBS containing 0.1% Tween 20 (TBS-T) and 5% milk for 1 hr at 25°C and then incubated overnight at 4°C with the following primary antibodies: anti-phospho-p44/42 MAPK (Thr202/Tyr204), anti-phospho-Akt (Ser473), anti-Akt, anti-phospho-p38 MAPK (Thr180/Tyr182), anti-p38 MAPK (Cell Signaling Technology, Inc., Danvers, MA, USA), anti-phospho-ERα (Ser118) (Merck Millipore), anti-ERK2, anti-Bek (C-17), anti-ERα, anti-lamin B, anti-E-cadherin (Santa Cruz Biotechnology) and anti-tubulin (Sigma-Aldrich). Membranes were then incubated with horseradish peroxidase (HRP)-conjugated secondary antibody (Sigma-Aldrich) for 1 hr at 25°C. Bound antibody was detected by enhanced chemiluminescence detection reagents (Pierce Biotechnology Inc., Rockford, IL, USA), according to manufacturer's instructions. Tubulin served to estimate the protein equal loading. Densitometric analysis was performed with Quantity One Program (Bio-Rad Laboratories S.r.l., Segrate, MI, Italy).

### Subcellular fractionation

The extraction procedure was performed with the ProteoExtract Subcellular Proteome Extraction Kit (Calbiochem, San Diego, CA, USA), according to manufacturer's instructions for adherent-growing cells. The cytosolic, nuclear and membrane proteins fractions were immediately used or stored at −80°C for later use. The efficiency of fractionation was determined by immunoblotting using β-tubulin antibody for cytoplasm, lamin B antibody for nucleus and E-cadherin antibody for membranes.

### Cell transfection and dual luciferase reporter assay

MCF-7 cells were seeded onto 24-well plates at a density of 2 × 10^5^ cells/well and cotransfected with 1 μg of the 3× ERE TATA Luc (oestrogen-responsive element-containing luciferase reporter) construct (Addgene, http://www.addgene.org, plasmid 11354) [27] and 300 ng of the control pRL-TK plasmid (Promega Italia S.r.l., Milano, Italy) for normalization of transfection efficiency. The putative promoter region of the human FGFR2 gene (−1103 to +459 relative to the transcriptional initiation site) was amplified and inserted into a luciferase reporter vector, as previously described [26]. Transfections with ERE-luc or pKGFR construct were carried out in triplicate using Lipofectamine 2000 (Invitrogen) following manufacturer's instructions. Luciferase activities were determined with Dual Luciferase Reporter Assay System (Promega) 24 hrs after treatment, according to manufacturer's protocol.

### Pluronic F127 rheological studies

Pluronic F127 (PEO100-PPO65-PEO100; Sigma-Aldrich) was dissolved in water at 20% w/v and heated from 10 to 40°C, at a speed of 2°C/min. The values of G' (elastic modulus) and G'' (loss modulus) were recorded with a Haake Rheostress 300 (Thermo Fisher Scientific, Waltham, MA, USA) in the linear viscoelasticity range, using a parallel plate geometry, and plotted *versus* temperature.

### Pluronic F127 *in vitro* release test

Loaded hydrogels containing 4 mg of myoglobin or prednisolone in 2 ml of a 20% w/v solution of Pluronic F127 were introduced into a dialysis membrane bag (MWCO 3500 Da) and the end-sealed dialysis bag was incubated in 60 ml of release medium (distilled water) at 37°C and shaken at a speed of 50 r.p.m. At predetermined time intervals, 0.5-ml aliquots of the release media were withdrawn and replaced with an equal volume of fresh solution. The amount of model molecule released was quantified by UV (for myoglobin) or HPLC-reverse phase (for prednisolone). Cumulative release in percentage was plotted *versus* time.

### Animals

CD1 mice (Charles River Laboratories Italia s.r.l., Calco, LC, Italy) were accommodated in the animal facility of the Service for Biotechnology and Animal Welfare of the Istituto Superiore di Sanità, according to EU Commission Recommendation of 18 June 2007 on guidelines for the accommodation and care of animals used for experimental and other scientific purposes, with controlled lighting (12 hrs/day) and temperature (20–24°C), and were given food and water *ad libitum*. Forty female mice were anaesthetized by intraperitoneal injection of ketamine (100 mg/kg) and xylazine (10 mg/kg), and ovariectomized at 4 weeks of age to induce vaginal atrophy. They were given Altromin C1000 Fitoestrogen Free diet (Altromin, Lage, Germany) for all the duration of the experiments. After 30 days, they were divided into groups of eight mice each and locally treated by daily introducing in the vaginal canal, 25 μl of hydrogel loaded with KGF (15 or 30 ng/die) (3.2 × 10^−8^ and 6.3 × 10^−8^ M/die, respectively) or with E2 (30 ng/die or 1 μg/die) (4.4 × 10^−6^ and 1.5 × 10^−4^ M/die, respectively). A control group was represented by non-ovariectomized mice, and a group of ovariectomized mice was treated with hydrogel only. After 60 days of treatment, mice were killed and vaginas were immediately explanted, formalin fixed and processed for histological analysis.

### Histology

Serial sections of the vaginal mucosa obtained from mice were stained with haematoxylin/eosin or stained with periodic acid-Schiff (PAS) staining and counterstained with haematoxylin to evaluate glycogen deposits. The vertical distance between the bottom surface of cells in the basal layer and the apical surface of cells in the superficial layer, designated as thickness, and the ratio between the epithelial area and the length of the basal membrane were obtained with the aid of NIH Image J v1.56 (National Institutes of Health, Bethesda, MD, USA) from multiple measurements in three animals for each group, three fields (10× magnification) from each animal. The cell layers that covered five adjoining basal cells were also counted in the same specimens.

### Immunohistochemistry

Serial sections of the vaginal mucosa were processed using the avidin-biotin peroxidase-complex technique (DAKO, Glostrup, Denmark) and incubated with mouse monoclonal anti-A-rat-PCNA (clone P10, 1:4000; Abcam, Cambridge, UK), which recognizes proliferating cells.

### ELISA

Serum levels of E2 and KGF in treated mice were measured using standard ELISA kits (R&D Systems, Minneapolis, MN, USA) with a sensitivity ≥10 pg/ml.

### Statistical analysis

Each set of experiments was repeated at least in triplicate, and standard deviations were calculated. Two-tailed unpaired Student's *t*-test was used for statistical analysis, and *P* values less than 0.05 were considered statistically significant.

### Study approval

The use of vaginal mucosa biopsies for isolation of HVMs conformed to the guidelines of the 1975 Declaration of Helsinki, as revised in 2004, and was approved by the Institutional Review Board of the Department of Gynecologic-Obstetrical and Urologic Sciences of Sapienza University of Rome. Written consent was obtained from all participants prior to inclusion in the study. All animal experiments were notified to and authorized by the Ministry of Health.

## Results

### Proliferation *in vitro* on human vaginal epithelium

**Fig. 1:**
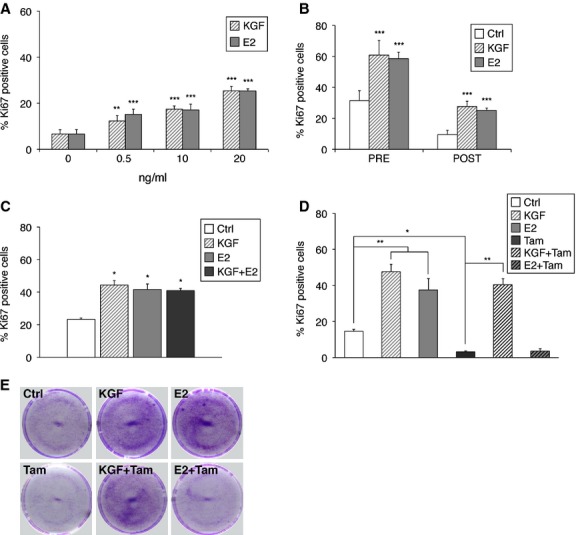
Effect of KGF and E2 on human vaginal mucosa cells (HVMs) proliferation. Immunofluorescence analysis with polyclonal antibodies against Ki67 was performed on: (**A**) HVMs treated with KGF (0.5, 10 or 20 ng/ml; 2.6 × 10^−11^, 1.1 × 10^−10^ and 1.1 × 10^−9^ M, respectively) or E2 (0.5, 10 or 20 ng/ml; 1.8 × 10^−9^, 7.3 × 10^−9^ and 7.3 × 10^−8^ M, respectively) for 24 hrs; (**B**) HVMs derived from biopsies of pre- or post-menopausal women treated with KGF (20 ng/ml) or E2 (20 ng/ml); (**C**) HVMs treated with KGF (20 ng/ml), E2 (20 ng/ml) or a combination of them for 24 hrs; (**D**) HVMs pre-treated with tamoxifen (Tam, 100 nM) for 30 min. and then treated with KGF (20 ng/ml), E2 (20 ng/ml), Tam, KGF plus Tam or E2 plus Tam for 24 hrs, which were also stained with 1% crystal violet (**E**). The percentage of Ki67-positive cells was determined by counting the number of Ki67-positive nuclei *versus* total number of nuclei in 10 different areas randomly taken from three different experiments. Error bars represent standard deviations. *P < 0.05; **P < 0.01; ***P < 0.001.

We first performed KGF and E2 treatments *in vitro* on HVMs obtained from biopsies of the vaginal mucosa from *n* = 3 healthy donors [25], to compare their effect on cell proliferation. Cell proliferation was determined by counting cells positive for Ki67 antigen, which identifies cycling cells, and reported in graph as percentage of positive cells (Fig. 1A). Our results show a dose-dependent increase in HVMs proliferation following treatment with both KGF and E2, as compared to untreated cells. To exclude an involvement of patients' age in the extent of proliferative response to KGF or E2 treatments, we performed the proliferation assays separately on HVMs obtained from *n* = 10 donors in pre-menopausal age and *n* = 10 in post-menopausal age. As shown in Figure 1B, despite a difference in the basal proliferation levels of untreated cells, as expected, KGF and E2 exerted their proliferative potential in both pre- and post-menopause-derived cells. These results indicate that KGF is able to stimulate the proliferation of vaginal epithelium *in vitro* as well as estradiol.

We also analysed the combined effect of KGF and E2, but we did not obtain synergistic or additive effect with respect to single treatments (Fig. 1C). To assess if the lack of synergy was because of the use of saturating amounts of KGF and E2, we also performed the same proliferation assay using various combinations of KGF and E2 at lower doses. As shown in Figure S1, we failed to obtain significantly synergistic or additive effect even with low doses of KGF and E2.

We then subjected HVMs to a pre-treatment with tamoxifen, a chemotherapeutic drug that is known to abolish oestrogen-mediated proliferation and to promote apoptosis, followed by treatment with KGF or E2. The data obtained, both by immunofluorescence with anti-Ki67 (Fig. 1D) and by staining with Crystal Violet (Fig. 1E), showed that the proliferative effect of E2 on HVMs was fully inhibited by the presence of tamoxifen, and cell viability was greatly impaired, as expected. Conversely, KGF was able to stimulate HVMs proliferation and to restore cell viability even in the presence of tamoxifen (Fig. 1D and E). These data suggest that KGF may be effective in the treatment of vaginal disorders also in patients undergoing cancer chemotherapy with tamoxifen for the treatment of oestrogen-sensitive tumours, which implies the ineffectiveness of oestrogen treatment.

### Analysis of the activation of signalling pathways by E2 and KGF

**Fig. 2:**
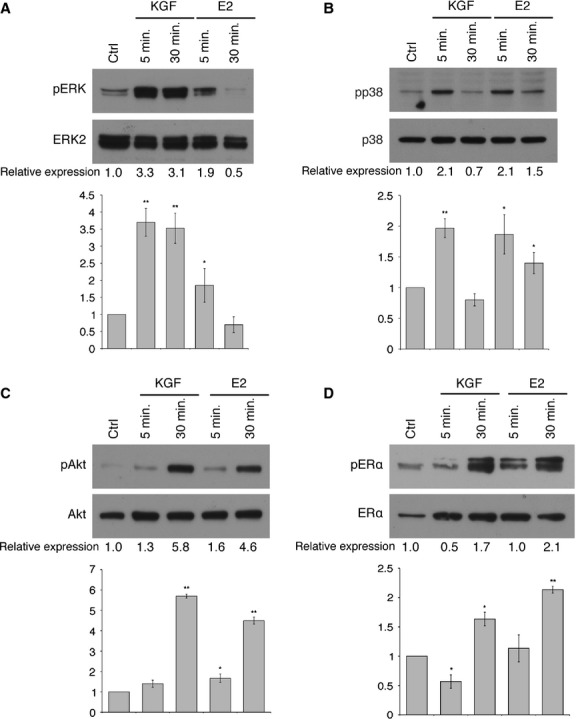
Effect of KGF and E2 on the activation of ERα non-genomic pathways. MCF-7 cells were treated with KGF (20 ng/ml) (1.1 × 10^−9^ M) and E2 (20 ng/ml) (7.3 × 10^−8^ M), for 5 or 30 min. (**A**) Western blot analysis with a phospho-specific ERK monoclonal antibody (pERK; Thr202/Tyr204). Levels of total ERK were assessed by blotting with an ERK2-specific antibody. (**B**) Western blot analysis with a phospho-specific p38 monoclonal antibody (pp38; Thr180/Tyr182). Levels of total p38 were assessed by blotting with a p38-specific antibody. (**C**) Western blot analysis with a phospho-specific Akt monoclonal antibody (pAkt; Ser473). Levels of total Akt were assessed by blotting with an Akt-specific antibody. (**D**) Western blot analysis with a phospho-specific ERα monoclonal antibody (pERα; Ser118). Levels of total ERα were assessed by blotting with an ERα-specific antibody. The images are representative of at least three independent experiments. The intensity of the bands was evaluated by densitometric analysis, normalized and reported in graphs as relative expression with respect to untreated cells (Ctrl). Error bars represent standard deviations. *P < 0.05, **P < 0.01.

We then analysed the activation of potential non-genomic ER pathways in MCF-7 cells, expressing both KGFR and ERα. We found a consistent activation of MAPK ERK1 and 2 after 5 and 30 min. of treatment with KGF. As for E2, we observed a less consistent phosphorylation of ERK1 and 2, restricted to only 5 min. of treatment (Fig. 2A). Both treatments were able to consistently phosphorylate the p38 protein within 5 min. (Fig. 2B) and to induce Akt phosphorylation, especially at 30 min., with KGF slightly more efficient than E2 (Fig. 2C). These data underscore the existence of an upstream crosstalk between the two pathways, thus suggesting a potential cooperation between KGF and E2 at the plasma membrane level.

Moreover, treatment with KGF at 30 min. was also able to induce a ligand-independent phosphorylation of ERα at Ser118 (Fig. 2D), thus prompting a direct role of KGF in ER activation.

As a control, we evaluated MAPK ERK 1/2 and Akt activation also in HVMs cells. As shown in Figure S2A, we observed a consistent phosphorylation of ERK1 and 2 after 5 and 30 min. of treatment with KGF and E2. Akt activation was observed especially at 30 min. of treatment with both KGF and E2 (Fig. S2B). To verify the capacity of KGF to phosphorylate Akt without oestrogen cooperation, we performed Western blot analysis of Akt phosphorylation status on the ERα-negative cell line MDA-MB-231, showing that KGF was able to phosphorylate Akt after both 5 and 30 min. of treatment (Fig. S2C).

**Fig. 3:**
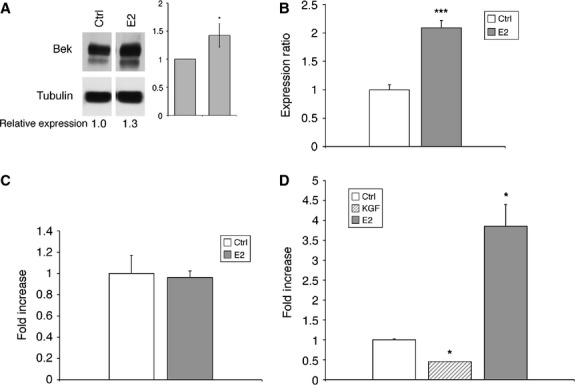
Evaluation of E2-mediated up-regulation of KGFR and KGF-mediated ERα transcriptional activation. (**A**) MCF-7 cells were treated or not with E2 (20 ng/ml) (7.3 × 10^−8^ M) for 24 hrs, and the amount of KGFR protein was evaluated by Western blot analysis with anti-bek polyclonal antibodies. Tubulin served as a loading control. The images are representative of at least three independent experiments. The intensity of the bands was evaluated by densitometric analysis, normalized and reported in a graph as relative expression with respect to untreated cells (Ctrl). (**B**) The levels of KGFR mRNA expression in E2-treated cells were determined by qRT-PCR, normalized to 18S mRNA levels and expressed as fold increase with respect to untreated cells (Ctrl). (**C**) The pKGFR construct was transfected into MCF-7 cells untreated or treated with E2 (20 ng/ml) for 24 hrs, and luciferase activities were determined. (**D**) The ERE-Luc construct was transfected into MCF-7 cells untreated or treated with KGF (20 ng/ml) (1.1 × 10^−9^ M) or E2 (20 ng/ml) (7.3 × 10^−8^ M) for 24 hrs, and luciferase activities were determined. Luciferase reporter assay data are expressed as fold increase with respect to untreated cells (Ctrl) and represent the mean of three separate experiments after correcting for differences in transfection efficiency by pRL-TK activities. Error bars represent standard deviations. *P < 0.05, ***P < 0.001.

To assess if oestrogens may affect the KGFR pathway, we carried out analyses of KGFR expression in MCF-7 cells treated with E2. The results obtained by Western blot indicated an increase in the amount of KGFR protein induced by E2 (Fig. 3A). qRT-PCR confirmed an up-regulation of KGFR mRNA after treatment with E2 (Fig. 3B). To verify whether E2-mediated KGFR up-regulation could involve a direct transcriptional activation, we cloned the putative FGFR2 gene promoter in a luciferase reporter vector and set up a transactivation assay. The plasmid harbouring the FGFR2 promoter was transfected in MCF-7 cells. As shown in Figure 3C, no significant increase in luciferase activity was observed following treatment with E2. Therefore, we can suggest that E2 regulation of KGFR expression is not mediated by direct activation of FGFR2 promoter.

Genomic ERα pathway implies that ERα, once activated by E2, is rapidly translocated to the nucleus, where it can directly stimulate the transcription of its target genes by binding to EREs. To test whether KGF was also able to stimulate this genomic pathway, we performed a transactivation assay with a plasmid containing a sequence of three EREs linked to the luciferase gene. E2, as expected, was able to determine the construct activation, while treatment with KGF even reduced basal construct activation (Fig. 3D).

**Fig. 4:**
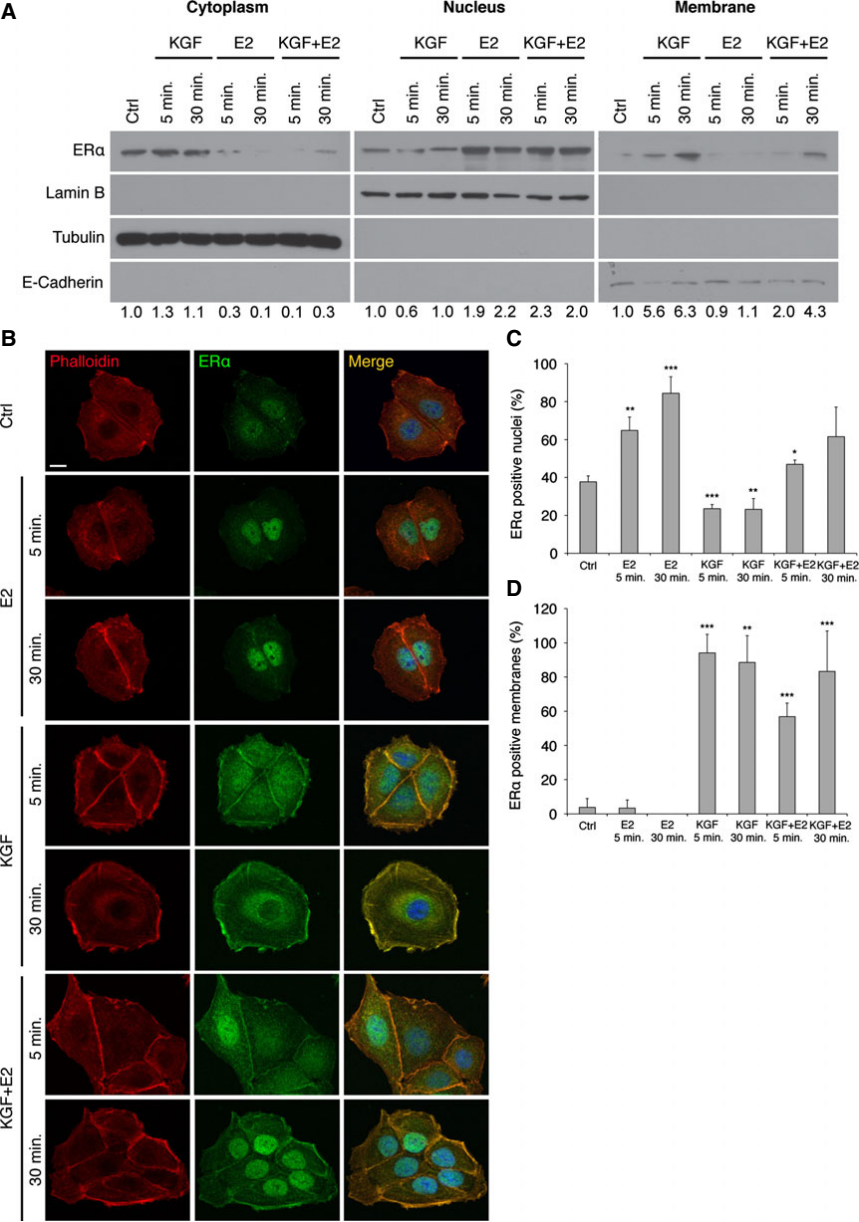
Subcellular ERα localization in MCF-7 cells. Cells were treated with KGF (20 ng/ml; 1.1 × 10^−9^ M), E2 (20 ng/ml; 7.3 × 10^−8^ M) or a combination of them for 5 or 30 min. (**A**) The cytoplasmic, nuclear and membrane fractions were analysed with anti-ERα, anti-β-tubulin, anti-lamin B and anti-E-cadherin antibodies. The intensity of the bands was evaluated by densitometric analysis, normalized and reported as relative expression with respect to untreated cells (Ctrl). (**B**) Cells were stained using anti-ERα primary antibodies followed by FITC-conjugated secondary antibody (green). Actin microfilaments were stained with TRITC-conjugated phalloidin (red). Merged images are shown (scale bar 10 μm). (**C** and **D**) Quantification of the results in **B**. Cells exhibiting primarily nuclear localization of ERα were counted and reported in graph as percentage of ERα-positive nuclei (**C**), while cells exhibiting membrane localization of ERα were counted and reported in graph as percentage of ERα-positive membranes (**D**). More than 50 stained cells per microscopic field were counted. Mean values were obtained from measurements of three microscopic fields and reported in graphs. Error bars represent standard deviations. *P < 0.05; **P < 0.01; ***P < 0.001.

We then assessed the effect of both E2 and KGF on ERα localization by means of subcellular fractionation. Lysates of MCF-7 cells treated with E2, KGF or a combination of them, at various times, were divided into cytoplasmic, nuclear and membrane proteins fractions and subjected to Western blot with anti-ERα. The purity of fractions was confirmed by using proteins alternatively expressed in the cytoplasm (tubulin), in the nucleus (lamin B) and in the plasma membrane (E-cadherin) as a control. The results (Fig. 4A) showed that in the absence of treatment ERα was distributed in the cytoplasmic and nuclear fractions, whereas right after 5 min. of treatment with E2, there was a dramatic redistribution of ERα in the nucleus, with a considerable reduction in its amount in the cytoplasmic fraction. Treatment with KGF, at the same dose and times, did not cause any significant redistribution of the receptor in the nucleus, but significantly increased the amount of ERα in the membrane fraction. Treatment with a combination of KGF and E2 gave a mixed result, with a distribution of ERα both at the plasma membrane and in the nucleus. We performed subcellular fractionation also in HVMs, obtaining similar results (Fig. S3).

To confirm these results, we carried out immunofluorescence microscopy with Apotome module, which allowed us to obtain optical sections and to identify the localization of multiple signals (Fig. 4B). We were able to detect the displacement of ERα in the nucleus after treatment with E2, as previously demonstrated. Moreover, we confirmed the unexpected localization of ERα on the plasma membrane after treatment with KGF, both alone and in combination with E2. Our data demonstrated that E2 was able to increase the percentage of ERα-positive nuclei (Fig. 4C), while KGF caused an increase in ERα-positive membranes (Fig. 4D), and treatment with both KGF and E2 reflected in an increase in both nuclear and membrane ERα. Therefore, it is conceivable that KGF is able to push ERα towards the non-genomic pathway, by preventing or at least delaying its nuclear translocation.

### Effect of E2 and KGF *in vivo* in an animal model of ovariectomized mice

**Fig. 5:**
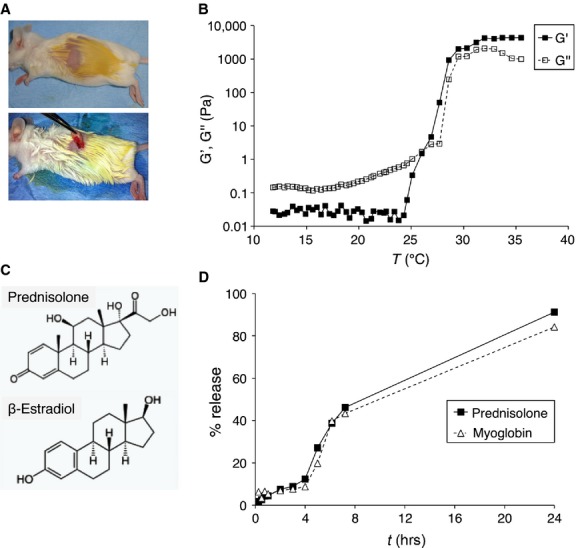
*In vivo* studies on a murine model. (**A**) CD1 mice were subjected to ovariectomy, to induce vaginal atrophy, and then locally treated daily with 25 μl of hydrogel loaded with KGF (15 or 30 ng/die; 3.2 × 10^−8^ and 6.3 × 10^−8^ M/die, respectively) or with E2 (30 ng/die or 1 μg/die; 4.4 × 10^−6^ and 1.5 × 10^−4^ M/die, respectively). (**B**) The values of G' (elastic modulus) and G'' (loss modulus) of a solution of Pluronic F127 (cP = 20% w/v in H_2_O) heated at the speed of 2°C/min., from 10 to 40°C were measured and reported as a graph. (**C**) Schematic diagrams showing the structural similarity of E2 and the model molecule used for Pluronic F127 *in vitro* release test (prednisolone). (**D**) The release profiles of hydrogels loaded with myoglobin or prednisolone were expressed in terms of cumulative release in percentage and plotted *versus* time (in hours).

The effect of local administration of KGF on vaginal atrophy was assessed *in vivo* on an animal model, represented by CD1 strain female mice. The study was conducted on a total of 50 mice aged 1 month. Female mice, *n =* 10 were left untreated, while *n* = 40 were subjected to ovariectomy (Fig. 5A). After 30 days from ovariectomy, we checked the fallen of E2 concentration in blood circulation.

We selected Pluronic F127, a synthetic polymer amphiphilic block, as a vehicle to allow the release of both KGF and E2. If solubilized in water at high concentrations (c ≥15% w/v), it is liquid at low temperatures, thus allowing homogeneous dilution of substances, but switches to the density of a hydrogel at higher temperatures (T >25°C), thus allowing its local application. We first demonstrated the liquid–gel transition that occurs with the increase in temperature, assessed by values of G' (elastic modulus) higher than those of G'' (loss modulus; Fig. 5B). Then we assessed the capability of releasing both hydrophilic proteins and lipophilic hormones, and their release kinetics, by employing two model molecules: myoglobin (MGB), a hydrophilic protein with a MW comparable to that of KGF (MGB = 16.7 kD, KGF = 19 kD); and prednisolone, a lipophilic hormone characterized by a chemical structure similar to that of estradiol (Fig. 5C). Hydrogels loaded with an exact amount of template molecules were placed into vials capped with a mesh panel and warmed at 37°C. The vials were inserted in the release media and percentage of release was assessed periodically. As shown in Figure 5D, the release curves of the two model molecules were almost identical, and the release was almost complete within 24 hrs, thus indicating the need of daily treatment.

Then, ovariectomized mice were divided into groups of eight each. One group received the vehicle only (OV); the other groups received, respectively: the vehicle plus E2 at 30 ng/day (4.4 × 10^−6^ M/die; E2 low); the vehicle plus E2 at 1 μg/day (1.5 × 10^−4^ M/die; E2 high); the vehicle plus KGF at 15 ng/day (3.2 × 10^−8^ M/die; KGF low); the vehicle plus KGF at 30 ng/day (6.3 × 10^−8^ M/die; KGF high). The group of not ovariectomized mice represented the control (Ctrl). At the end of daily treatments, which lasted 60 days, the animals were killed. Prior to killing, blood samples were collected from all animals, to assess the serum levels of E2 and KGF by specific ELISA assays. As reported in Table 1, KGF did not pass the barrier of cell membranes and was not released into the bloodstream, unlike E2 that reached the systemic circulation already with low-dose treatment and more consistently after high-dose treatment.

**Table 1:** ELISA assays for detection of KGF and E2 in the serum of treated mice

Group	E2 (pg/ml)	KGF (pg/ml)
Ctrl	123.6 ± 17.6*	ND†
OV	ND	ND
E2 low	20.3 ± 0.6	ND
E2 high	202.7 ± 9.1	ND
KGF low	ND	ND
KGF high	ND	ND

* Mean ± SD.

† Not detectable.

**Fig. 6:**
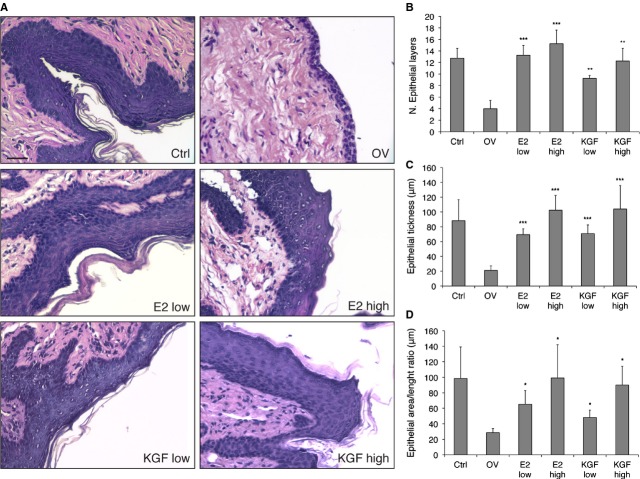
Effect of KGF and E2 *in vivo* on vaginal epithelium. (**A**) Tissue sections of the vagina of control mice (Ctrl), ovariectomized mice treated with vehicle only (OV) and mice treated with low and high doses of E2 or KGF were stained with haematoxylin/eosin. Representative tissue sections for each treatment group are shown (scale bar 50 μm). (**B**–**D**) The number of epithelial layers (**B**), the thickness of epithelial cells (**C**) and the ratio between the epithelial area and the length of the basal membrane (**D**) were calculated for each treatment. Briefly, three animals for each group were processed and three images were taken from each animal. Mean values were obtained from five measurements of each image. Error bars represent standard deviations. *P < 0.05; **P < 0.01; ***P < 0.001.

Immediately after killing, the vaginas were removed, fixed in formalin and analysed histologically by means of haematoxylin/eosin (Fig. 6A). Vaginal epithelium of Ctrl group showed stratified squamous epithelium with cornified layer and presence of a well-formed *stratum lucidum*. In the OV group, vaginal mucosa showed a significant decrease in epithelial thickness in comparison with Ctrl. A significant induction in vaginal epithelium stratification, hyperkeratosis and cornification was observed in the vaginas exposed to both E2 and KGF (especially in the high-dose treatment groups). In OV animals, there was a significant thinning of the epithelium, consisting of approximately four layers of cells. On the other hand, in E2- and KGF-treated animals, the epithelium presented 13–15 and 9–12 cellular layers, respectively (Fig. 6B). These findings were confirmed by histomorphometric analysis, showing a significant increase in epithelial thickness and of the ratio between area of the epithelial layer and length of the basement membrane in both E2-high and KGF-high groups when compared with OV group (Fig. 6C and D). In conclusion, epithelial thickness after treatment with KGF was comparable to that of Ctrl, and comparable or slightly lower than that obtained with E2 treatment.

**Fig. 7:**
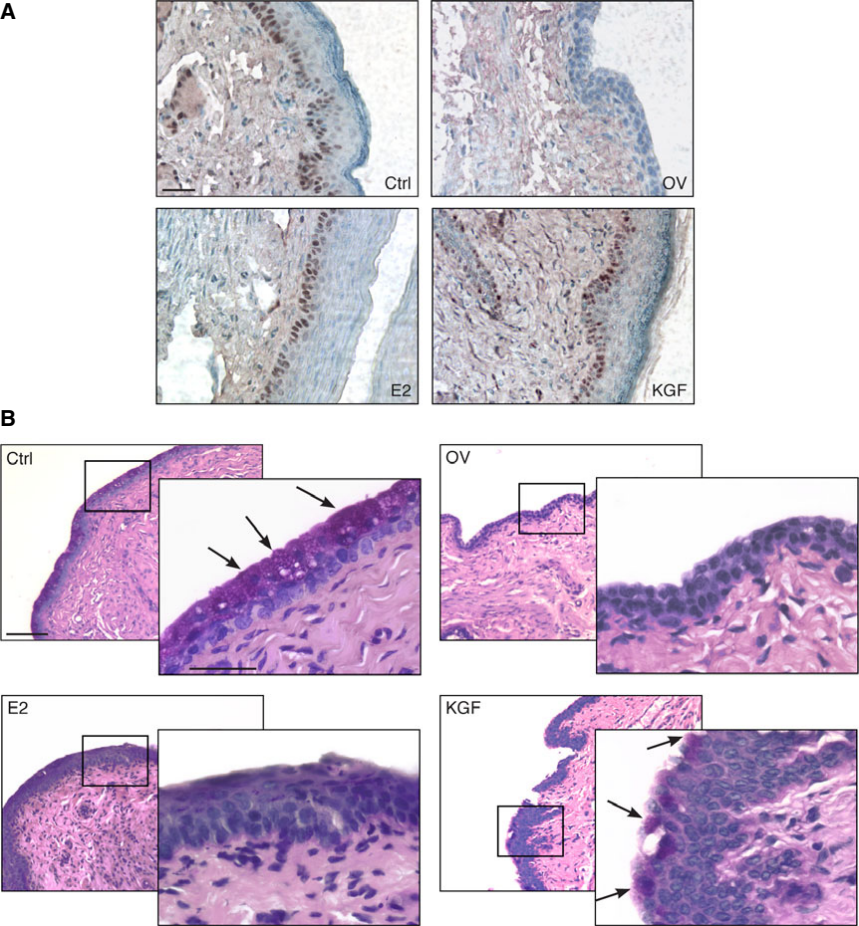
Characterization of the vaginal epithelium after treatment with KGF and E2. (**A**) Tissue sections were subjected to immunohistochemistry with an anti-PCNA antibody to assess basal cells proliferation. Sections were counterstained with haematoxylin. Representative tissue sections for each treatment group are shown (scale bar 50 μm). (**B**) Tissue sections were subjected to periodic acid-Schiff (PAS) staining procedures. Representative tissue sections for each treatment group are shown (scale bar 100 μm). A significant area in each panel is indicated by a square and an enlargement of this area is shown in the overlying panel (scale bar 50 μm). Arrows indicate PAS-positive cells.

We also assessed the proliferative capacity of keratinocytes through immunohistochemistry with PCNA, a marker of cell proliferation. As documented in Figure 7A, both KGF and E2 treatments were able to restore basal cell proliferation, abolished by ovariectomy. Furthermore, we analysed vaginal sections after PAS staining procedure, which stains all 1,2-glycol carbohydrates (glycogen, mucins and glycolipids; Fig. 7B). Ctrl mice revealed the presence of mucified cells in the apical surface of the epithelium, characterized by the presence of secretory vesicles and positive PAS staining (Fig. 7B, arrows). A dramatic decrease in the appearance and extent of mucified epithelium was observed in OV animals, where no PAS staining was detected. E2 treatment induced a thickened epithelium, consisting of slightly keratinized cells rather than mucified cells, probably because of the role of E2 in the post-translational modifications of keratins in the apical layer of the vaginal epithelium [28]. Conversely, KGF treatment partially retrieved the mucified epithelial morphology, thus suggesting that it could contribute to restore the physiological secretion of mucosubstances in the atrophic vaginal epithelium (Fig. 7B, arrows).

## Discussion

It is known that oestrogens are regulators of growth and differentiation in a wide range of tissues, including reproductive organs, mammary gland, and nervous, cardiovascular and skeletal systems. Our expertise in isolating and culturing epithelial cells derived from vaginal mucosa biopsies [25] allowed us to assess E2 proliferative effect on this tissue and to compare it with KGF, a known growth factor with specific mitogenic activity for epithelial cells.

Vaginal symptoms, including dysuria, pain, mucosal atrophy and vaginal drying, represent a significant health concern for the female population caused by oestrogen deficiency occurring at menopause or because of local treatments for endometrial cancer, such as vaginal brachytherapy [1,4]. The most widely used remedy for vulvo-vaginal disorders is systemic HRT. However, involvement of oestrogens in arising-progression of endometrial, ovarian and breast cancers created distrust of women and also of some physicians in recommending adjuvant therapy [15,16,29]. Up to now, no consensus has been achieved with regard to appropriate therapy: HRT should be given to women with menopausal complaints to meet their needs, taking into account their individual risk profile and the overall therapeutic objectives. Intravaginal oestrogen formulations have been introduced to avoid systemic exposure to oestrogens and are now preferred in women with no other menopausal symptoms requiring systemic treatment [9,30]. However, the safety of local oestrogen treatment has been discussed, as data about the potential increase in serum levels of oestradiol with the use of vaginal tablets and creams are controversial. Despite some reports indicated that vaginal administration of oestrogens exerts mainly a local effect, with limited changes in systemic effect [31], other studies demonstrated that intravaginal oestrogen preparations cause a significant increase in serum oestradiol, thus indicating that their effects are not limited to the vagina, but can determine also systemic actions [8,9]. Such observations have been also confirmed by our results (Table 1). In the light of these considerations, non-hormonal preparations have been developed to treat atrophic vaginitis, such as soy-derived isoflavones [32]. Nevertheless, the use of these topic formulations has yielded poor results in terms of effectiveness [33,34]. Therefore, our proposal of an alternative compound to treat atrophic vaginitis might contribute to overcome the problem of considering the controversial data about the safety of vaginal oestrogen therapy.

Our study was designed to compare the *in vivo* effects of E2 and KGF local treatment. We took advantage from the existence of Pluronic F127, a polymer already widely used in the biomedical field (wound healing, tissue engineering, gene delivery) [35] because of its capability to change density in solution depending on the temperature and to release both hydrophilic substances and lipophilic hormones with similar kinetics. Here, we demonstrated that local administration of KGF can be advantageous not only for its efficacy in stimulating vaginal cells proliferation, but also as it does not cross the membrane barrier, it is not released into the bloodstream and, therefore, topic treatment is completely devoid of systemic effects. Moreover, recently the researchers underlined the need for further studies to explore the risk of breast cancer recurrence after vaginal oestrogen application in oncological patients [36]. In the light of these considerations, KGF positive effect on the viability of tamoxifen-treated cells, previously demonstrated by our group on breast cancer cell lines [37] and now confirmed also on HVMs, suggests that the administration of KGF for the relief of vaginal symptoms can be even more effective than the use of oestrogens, especially in oncological patients treated with tamoxifen or other chemotherapeutic drugs at anti-oestrogenic action.

The biological effects of oestrogens are mainly mediated by binding and activation of ERα, which regulates transcription by interacting with EREs within the promoter of target genes. In addition, oestrogens may mediate signals initiated at the plasma membrane, which trigger a rapid intracellular transduction, thus activating non-genomic pathways such as that of MAPK or PI3K/Akt [10].

Interestingly, oestrogen pathway may also affect growth factor receptors signalling, such as those of EGFR and IGF-IR [14,15]. In particular, in human prostate cancer cells, E2 is able to induce a significant up-regulation of IGF-IR protein and mRNA expression, thus strengthening IGF-I biological effects in terms of cell proliferation, migration and resistance to apoptosis [38]. Furthermore, both EGF and IGF-1 are able to determine the phosphorylation of ERα at Ser118 and to stimulate its transcriptional activity [39]. Moreover, it has been previously reported that KGF-induced proliferation in MCF-7 cells can be significantly reduced by siRNA inhibition of ERα, thus suggesting the existence of an overlapping between oestrogen and KGFR signalling [40].

In this study, we showed that E2 treatment was able to increase both KGFR protein and mRNA expression in MCF-7 cells, although it did not induce a direct transcriptional activation on KGFR promoter. A possible explanation for this observation is that oestrogen regulatory activity could be mediated by modulation of miRNAs rather than by direct binding to KGFR promoter. However, further studies are needed to address the mechanisms involved in the modulation of KGFR expression induced by E2.

Activation of ERα nuclear signalling, because of its effect on gene expression [41,42], has been indicated as the main player of E2 systemic effects, such as the increased incidence of breast and endometrium cancers. In fact, although non-nuclear ER activation is able to stimulate ERK phosphorylation in both endometrial cells and MCF-7 cells, some reports demonstrated that this unique event is not sufficient to stimulate the growth of cancer cells in culture or *in vivo*, therefore it is not able to promote breast cancer tumour growth or uterine carcinogenesis [41,43].

Interestingly, we showed that KGF does not stimulate oestrogen genomic pathway, as demonstrated by its failure to activate ERE-luc expression. On the contrary, our results suggest that KGF could even prevent the activation of this pathway by retaining ERα at the plasma membrane, thus avoiding its nuclear translocation. The different effect of KGF and E2 on ERα distribution could also account for the lack of synergistic or additive effect of these substances on cell proliferation, which cannot be ascribed to the use of saturating doses as it was observed also at low doses of KGF and E2.

Nevertheless, we observed that KGF is able to activate the same protein kinases activated by ERα non-genomic pathway (p38, ERK and PI3K/Akt) at both early and late time-points, and to stimulate proliferation in HVMs. In particular, we observed that Akt pathway is activated by KGF in an oestrogen-independent manner, as demonstrated by Akt phosphorylation after KGF treatment also in ERα-negative cells. Such result is in accordance with previous data indicating both KGF ability to phosphorylate Akt in non-oestrogen-sensitive cells, such as the immortalized keratinocytes HaCaT [44] and the ability of oestrogen itself to activate the PI3K-Akt signalling through an ER-independent mechanism [45].

Taken together, our results highlight the existence of an interplay between oestrogen and KGFR pathways, and contribute to identify the molecules involved in such interaction, thus clarifying the mechanisms underlying the growth-promoting effect of oestrogens. Moreover, we observed that treatment with KGF *in vitro* was able to alter the intracellular distribution of ERα, thereby influencing also the choice between oestrogen genomic and non-genomic signalling pathway. The evidence that topic administration of KGF is as efficient as local oestrogen treatment for the recovery of vaginal atrophy in ovariectomized mice provides a rationale for its pre-clinical development in humans, and a patent has been applied (no. RM2012A000404). We are currently developing alternative delivery methods, such as vaginal rings or tablets, to provide sustained KGF release into the vaginal lumen and to prolong the period of time between treatments. As clinical application of KGF has been approved from FDA for the treatment of patients with haematological or head and neck cancers to prevent oral mucositis [21,22], and considering the beneficial effect of KGF on vaginal mucosa illustrated in the present study, we believe that clinical trials testing this strategy in humans are sufficiently warranted, and we hope that it could be readily translated into therapy to improve the quality of life for post-menopausal women.

## Supporting information

**Figure S1** Effect of different doses of KGF and E2 alone or in combination on HVMs proliferation.

**Figure S2** Effect of KGF and E2 on the activation of ERα non-genomic pathways in other cell lines.

**Figure S3** Subcellular ERα localization in HVMs.
