# Supplementary figures and images for: Topical KGF treatment as a therapeutic strategy for vaginal atrophy in a model of ovariectomized mice
Source: J Cell Mol Med. 2014 Aug 1;18(9):1895–907. doi: 10.1111/jcmm.12334 (PMC4196664; doi:10.1111/jcmm.12334)

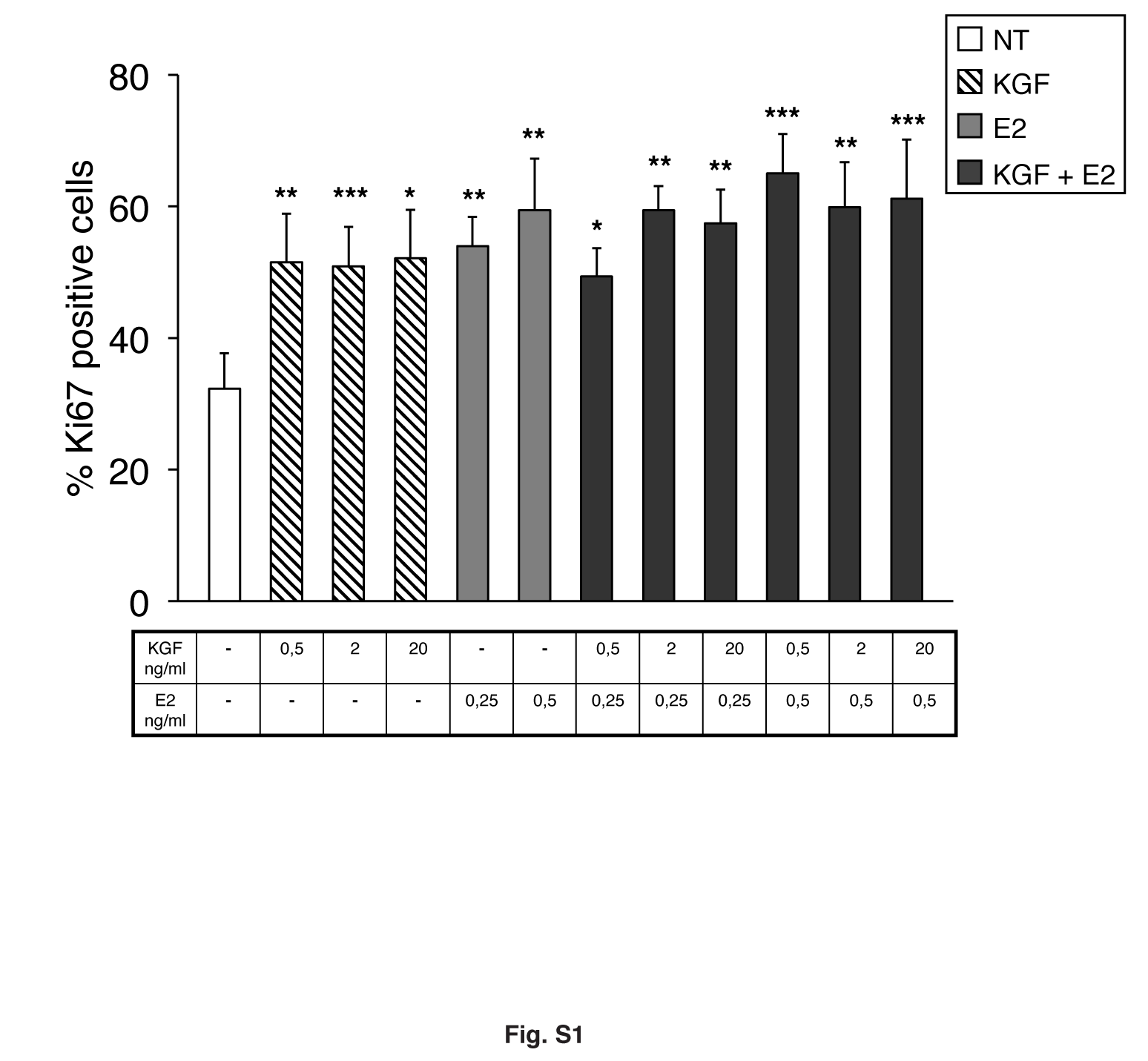

Supplement: Supplementary file 1 — Figure S1 Effect of different doses of KGF and E2 alone or in combination on HVMs proliferation. [file jcmm0018-1895-SD1.tif]

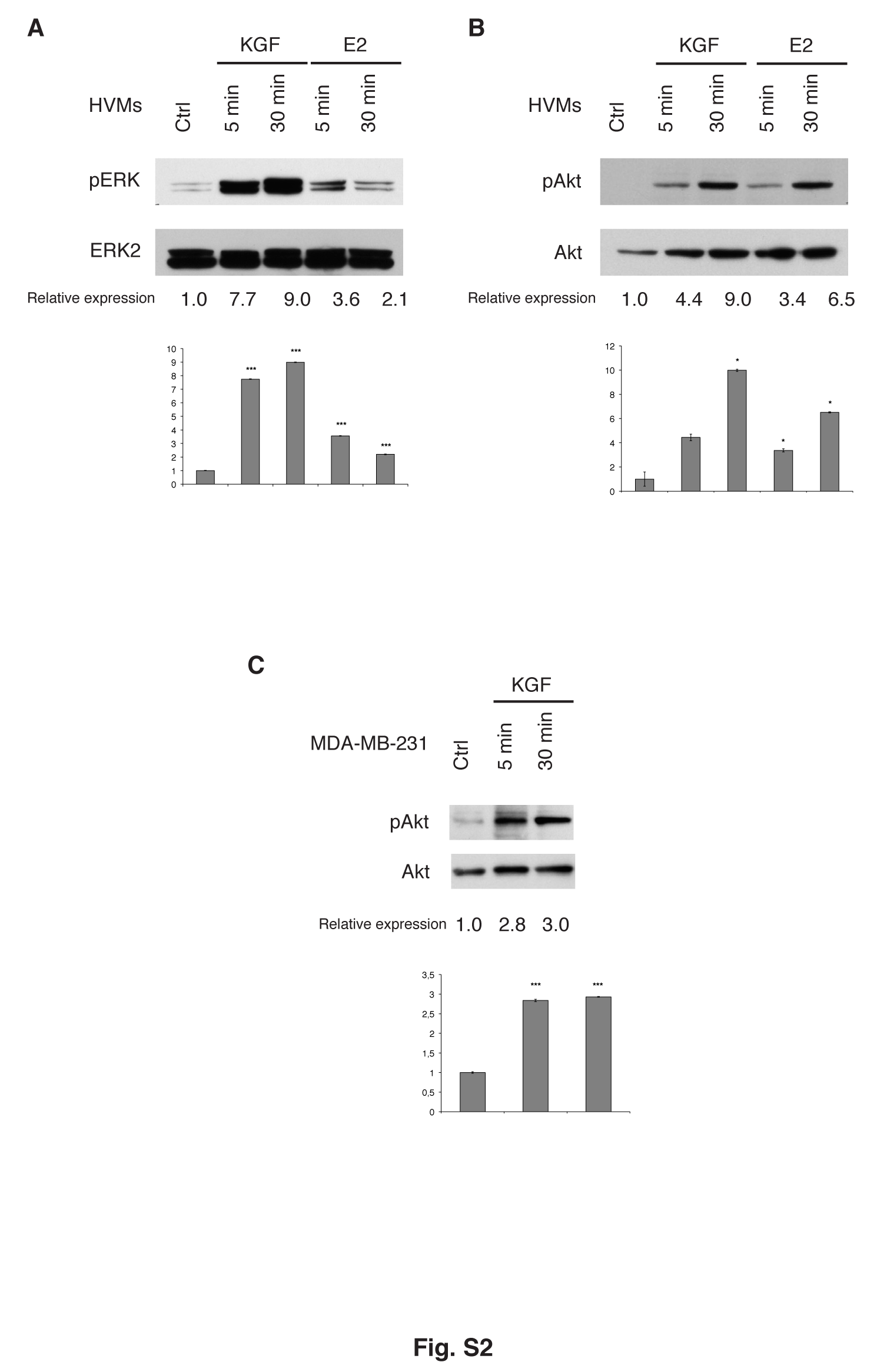

Supplement: Supplementary file 2 — Figure S2 Effect of KGF and E2 on the activation of ERα non-genomic pathways in other cell lines. [file jcmm0018-1895-SD2.tif]

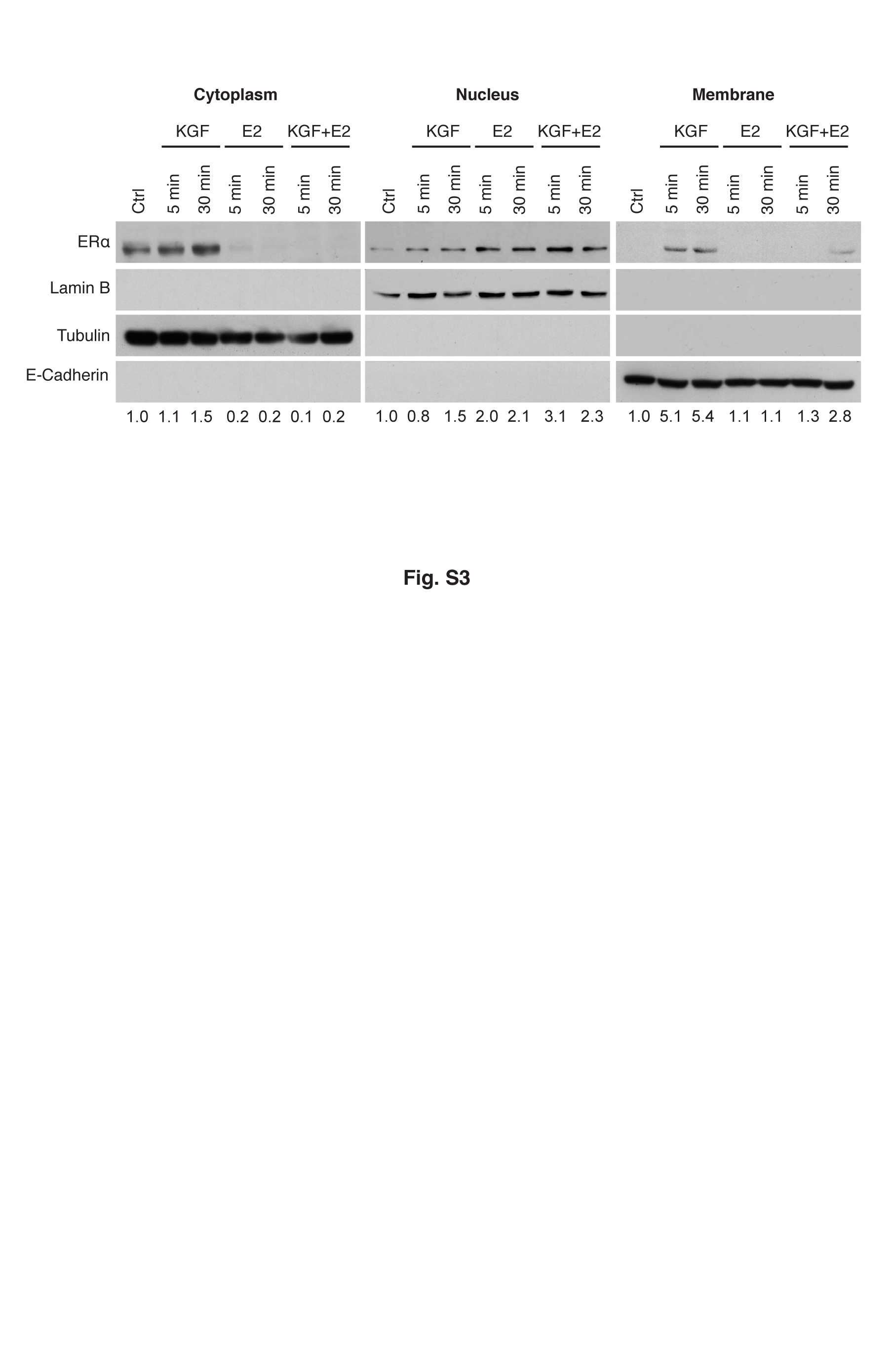

Supplement: Supplementary file 3 — Figure S3 Subcellular ERα localization in HVMs. [file jcmm0018-1895-SD3.tif]
