# Supplementary material for: Topical KGF treatment as a therapeutic strategy for vaginal atrophy in a model of ovariectomized mice
Source: J Cell Mol Med. 2014 Aug 1;18(9):1895–907. doi: 10.1111/jcmm.12334 (PMC4196664; doi:10.1111/jcmm.12334)
Supplement: Supplementary file 4 — Figure S3 Subcellular ERα localization in HVMs. [file jcmm0018-1895-SD4.pdf]

## SUPPORTING INFORMATION

## MATERIALS AND METHODS

### Cell cultures and treatments

The ERα-negative MDA-MB-231 human breast adenocarcinoma cell line, purchased from the American Type Culture Collection (No. HTB-22, ATCC-LGC Promochem, Teddington, UK), were cultured in Dulbecco's Modified Eagle's Medium (DMEM; Invitrogen, Karlsruhe, Germany), supplemented with 10% fetal bovine serum (FBS; Invitrogen) and antibiotics.

## FIGURE LEGENDS

**Figure S1. Effect of different doses of KGF and E2 alone or in combination on HVMs proliferation.** Immunofluorescence analysis with polyclonal antibodies against Ki67 was performed on HVMs treated with KGF (0.5, 2 or 20 ng/ml) ($2.6x10^{-11}$, $1.1x10^{-10}$ and $1.1x10^{-9}$ M, respectively), E2 (0.25 or 0.5 ng/ml) ($9.2x10^{-10}$ and $1.9x10^{-9}$ M, respectively) or a combination of them. The percentage of Ki67-positive cells was determined by counting the number of Ki67-positive nuclei versus total number of nuclei in ten different areas randomly taken from three different experiments. Error bars represent standard deviations. *P < 0.05; **P < 0.01; ***P < 0.001 *vs* NT.

**Figure S2. Effect of KGF and E2 on the activation of ERα non-genomic pathways in other cell lines.** HVMs (**A**, **B**) and MDA-MB-231 cells (**C**) were treated with KGF (20 ng/ml) ($1.1x10^{-9}$ M) or E2 (20 ng/ml) ($7.3x10^{-8}$ M) for 5 or 30 min. (**A**) Western blot analysis with a phospho-specific ERK monoclonal antibody (pERK) (Thr202/Tyr204). Levels of total ERK were assessed by blotting with an ERK2-specific antibody. (**B**, **C**) Western blot analysis with a phospho-specific Akt monoclonal antibody (pAkt) (Ser473). Levels of total Akt were assessed by blotting with an Akt-specific antibody. The images are representative of at least three independent experiments. The intensity of the bands was evaluated by densitometric analysis, normalized and reported in graphs as relative expression with respect to untreated cells (Ctrl). Error bars represent standard deviations. *P < 0.05;

**P < 0.001.

**Figure S3. Subcellular ERα localization in HVMs.** Cells were treated with KGF (20 ng/ml) ($1.1 \times 10^{-9}$ M), E2 (20 ng/ml) ($7.3 \times 10^{-8}$ M) or a combination of them for 5 or 30 min. The cytoplasmic, nuclear and membrane fractions were analyzed with anti-ERα, anti-β-tubulin, anti-lamin B and anti-E-cadherin antibodies. The intensity of the bands was evaluated by densitometric analysis, normalized and reported as relative expression with respect to untreated cells (Ctrl).
